# An AutoEncoder and LSTM-Based Traffic Flow Prediction Method

**DOI:** 10.3390/s19132946

**Published:** 2019-07-04

**Authors:** Wangyang Wei, Honghai Wu, Huadong Ma

**Affiliations:** 1 Beijing Key Laboratory of Intelligent Telecommunication Software and Multimedia, Beijing University of Posts and Telecommunications, Beijing 100876, China; 2 Information Engineering College, Henan University of Science and Technology, Luoyang 471023, China

**Keywords:** AutoEncoder, long short-term memory, traffic flow prediction

## Abstract

Smart cities can effectively improve the quality of urban life. Intelligent Transportation System (ITS) is an important part of smart cities. The accurate and real-time prediction of traffic flow plays an important role in ITSs. To improve the prediction accuracy, we propose a novel traffic flow prediction method, called AutoEncoder Long Short-Term Memory (AE-LSTM) prediction method. In our method, the AutoEncoder is used to obtain the internal relationship of traffic flow by extracting the characteristics of upstream and downstream traffic flow data. Moreover, the Long Short-Term Memory (LSTM) network utilizes the acquired characteristic data and the historical data to predict complex linear traffic flow data. The experimental results show that the AE-LSTM method had higher prediction accuracy. Specifically, the Mean Relative Error (MRE) of the AE-LSTM was reduced by 0.01 compared with the previous prediction methods. In addition, AE-LSTM method also had good stability. For different stations and different dates, the prediction error and fluctuation of the AE-LSTM method was small. Furthermore, the average MRE of AE-LSTM prediction results was 0.06 for six different days.

## 1. Introduction

In recent years, the increasing number of vehicles aggravates traffic congestion. The traffic congestion often brings a series of problems such as environmental pollution, which greatly reduces the quality of urban life. To improve the urban living environment, there are emerging fields such as smart cities and Internet of Things (IoTs) [1,2], which mainly utilize various information technologies to optimize urban resources and services. As an important part of smart cities, Intelligent Transportation Systems (ITSs) can effectively alleviate urban traffic congestion [3,4]. Hence, ITSs have received significant interest and have become one of the important development directions of modern transportation. The prediction of traffic flow plays a key role in ITSs. The normal operation of many large systems relies on accurate prediction of traffic information, such as the Split Cycle Offset Optimization Technique (SCOOT) system and Sydney Coordinated Adaptive Traffic (SCAT) system [5].

The early traffic flow prediction methods mainly include AutoRegressive Integrated Moving Averaging (ARIMA) [6], Kalman filter [7,8], Support Vector Machine (SVM) [9], Markov chain model [10], etc. These prediction methods are simple and can be implemented easily. However, these models cannot mine the deep relationship between data. For this reason, neural network models are used to predict the traffic flow since these models can mine big data and discover internal structure and potential characteristics. Recently, Deep Belief Network (DBN) [11,12], Long Short-Term Memory (LSTM) neural network [13,14,15] and prediction methods based on deep architecture [16,17] have been widely used in traffic flow prediction. Huang et al. [17] used the Gated Recurrent Unit (GRU) to predict the traffic flow, but this method only considers the temporal patterns of the traffic flow and ignores its spatial patterns. Because of the network structure of urban roads, the change of traffic flow in adjacent position is one of the important factors affecting traffic flow. Considering the temporal and spatial patterns of traffic flow, Lv et al. [16] exploited the deep learning architecture to predict traffic flow. However, compared with LSTM network, the deep learning model requires a large amount of data and computation. In addition, the LSTM network can effectively preserve the long-term effect of the data for time series data. Therefore, the LSTM network is a very promising prediction model for time series data.

In this paper, we consider the temporal and spatial patterns, and propose a prediction model, called AutoEncoder Long Short-Term Memory (AE-LSTM) prediction method. The AutoEncoder is used to obtain the traffic flow characteristics of adjacent positions. The adjacent positions represent the upstream and downstream location of the current location. We also use LSTM to predict the traffic flow at the current location. Compared with other prediction methods based on neural networks, AE-LSTM prediction model not only mines big data deeply, but also considers the influence of upstream and downstream traffic flow. Experimental results show that the proposed method has better performance on real datasets. The main contributions of this paper are as follows:

- We propose an AE-LSTM model to predict traffic flow. This method combines AutoEncoder with LSTM, where AutoEncoder is used for feature extraction and LSTM model is used for data prediction.
- We propose a traffic flow prediction algorithm based on AE-LSTM. The AutoEncoder and LSTM network are trained, respectively. Then, we fine-tune the whole network.
- We evaluated the performance of AE-LSTM by experiments. We conducted AE-LSTM on real datasets, and experimental results show that the performance of AE-LSTM was better than the other prediction methods.

The rest of this paper is organized as follows. In Section 2, we give a brief review of the related works of other researchers. In Section 3, we briefly introduce the AutoEncoder and LSTM, and then describe the AE-LSTM prediction model presented in this paper. In Section 4, we describe the implementation of the model in detail, including the training of the prediction network and data processing In Section 5, we provide the experiment results. In Section 6, we conclude the whole paper.

## 2. Related Work

The prediction of traffic flow is necessary for traffic plan and plays an important role in the mobile opportunistic networks [18,19,20]. For a long time, researchers have been working on traffic flow prediction and proposed many research methods. Early prediction models were very simple, such as random walks, historical averages [21], etc. Although these models are easy to implement, the prediction accuracy is not enough. In addition, people pay more and more attention to privacy protection [22], and many traditional prediction methods have difficulty guaranteeing privacy. Subsequently, many more complex and effective prediction models emerged.

Researchers usually use some time series models to make predictions, such as ARIMA, Markov chains, Gaussian processes, etc. Williams et al. [23] presented a multivariate ARIMA model containing upstream traffic data. Stathopoulos and Karlaftis [24] proposed a state space model of multiple time series, which uses upstream flow detector to obtain data. Kamarianakis and Prastacos [25] proposed a spatiotemporal autoregressive integrated moving average model to predict urban traffic flow. Min and Wynter [26] developed a time-based extension prediction method that takes into account the interaction between time and space. Kumar et al. [27] proposed a prediction scheme using the Seasonal ARIMA (SARIMA) model. This scheme predicts traffic flow through limited data. The authors used the difference method to stabilize the input data and the maximum likelihood method to estimate the model parameters. Pan et al. [28] introduced the spatial-temporal correlation to the short-term traffic flow prediction by using the random region transmission framework. Considering the interpretability of the prediction model, Xu et al. [29] proposed an interpretable spatial-temporal multiple adaptive bayesian regression model. Bayesian derivation is used to train model parameters. Because the traffic flow of adjacent sections affects each other, Sun et al. [30] constructed a bayesian network for traffic flow of each section to predict the traffic flow. However, when the traffic network is too complex, the accuracy of this method is not enough. Due to the dynamics and randomness of highway traffic, a short-term highway traffic prediction method based on the hidden Markov model [31] is proposed. This method uses the observed velocity statistics to define the traffic state in two-dimensional space. Xie et al. [32] used Gauss regression process to model and forecast traffic flow. Because of the interconnection between roads, traffic flow data are a kind of multivariable time series. Dynamic systems have good performance in simulating multivariable time series data. Therefore, Zhao and Sun [33] modeled the traffic flow according to the Gaussian process dynamic model. Then, a dynamic prediction model based on fourth-order Gaussian process is proposed, and the weighted neural network is introduced into the model to train the model parameters.

The Urban Traffic Control (UTC) systems and highway management systems put into use collect a large amount of traffic condition data every day. The big data in the field of transportation have attracted the interest of many scholars [34,35]. The previous prediction models cannot give full play to the advantages of big data, and have some shortcomings such as the lack of high accuracy and the inability to fully mine the historical information. In contrast, the neural network is very good at processing big data. Therefore, many prediction models based on neural networks are applied to traffic flow prediction. Tan et al. [36] proposed an aggregate prediction method based on neural network, which combines four prediction methods: neural network, ARIMA, exponential smoothing and moving average. The periodic similar time series are constructed from the original time series, and then the traffic flow is predicted by multiple prediction models. Tan et al. [11] proposed two DBN traffic prediction architectures based on Restricted Boltzmann Machines (RBMs). Lv et al. [16] exploited AutoEncoder model to forecast traffic flow. AutoEncoder is widely used in denoising processing, and can also be used as feature extractor. Chen et al. [37] used the deep learning model to predict the traffic flow in the case of special events, and proved that the RNN has good performance in traffic flow prediction. Considering the influence of weather conditions on traffic flow, Fu [14] proposed a forecast model combining RNN and GRU. GRU is a kind of RNN, but its cell structure is simpler than LSTM cell structure. Because GRU simplifies the structure of memory unit, some important information may be ignored in the prediction process, and the prediction accuracy is reduced. Similar to GRU, LSTM is also an improved RNN, which can retain the influence of data for a longer time, and improve the gradient disappearance for RNN. Therefore, LSTM is often used as a time series prediction model. Because future traffic conditions may be related to events that occurred long ago, Ma et al. [13] proposed a new LSTM neural network prediction model that can learn time series and automatically determine the prediction delay.

In addition, Yang et al. [38] proposed a new traffic state prediction method, which uses electrosleidicalography data and driving behavior to prediction traffic state. However, the data acquisition process is more complex in this prediction method. Wang et al. [39] used Error feedback Recurrent Convolutional Neural Network structure (eRCNN) to predict the continuous traffic. This deep network model introduces new error feedback neurons to better deal with emergencies. Wu et al. [40] proposed a traffic flow prediction model based on DNN. This model introduces an attention based model to determine the importance of past traffic flow and uses convolutional neural network to mine the spatial characteristics of traffic flow. Zhan et al. [41] combined a variety of prediction methods and proposed an automated framework to solve the problem of traffic flow prediction.

Different from many neural network prediction models, AE-LSTM prediction model uses AutoEncoder to extract the characteristic of upstream and downstream data, which can not only take into account the spatial characteristics of vehicle flow, but also not add too many calculations in the subsequent LSTM prediction. Meanwhile, LSTM network is used to explore the time characteristics of vehicle flow.

## 3. Methodology

### 3.1. AutoEncoder Model

**Figure 1:**
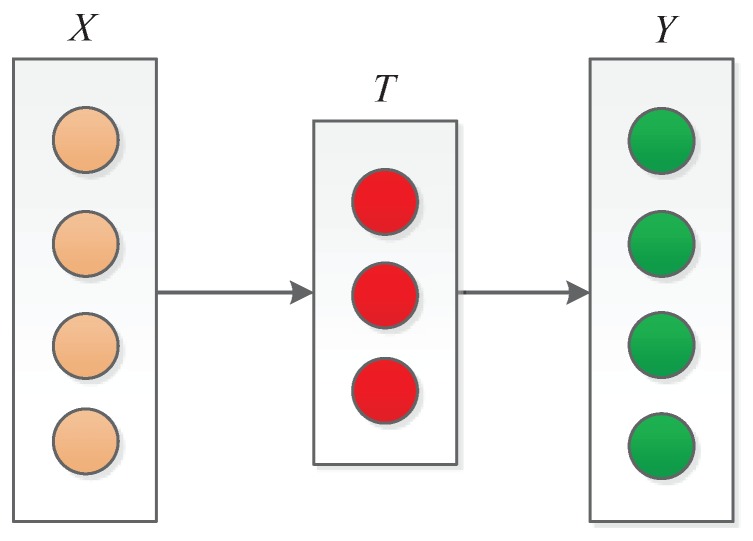
The structure of AutoEncoder. The encoder obtains the characteristic sequence *T* based on the original sequence *X*. The decoder gets the reconstructed sequence *Y* according to the characteristic sequence *T*.

AutoEncoder is usually used to reduce dimensions or extract features [42]. Given an original input sequence data $X=\{x_{1},x_{2},\ldots ,x_{k}\}$, where $x_{i}\in R^{d}$. The characteristic sequence of the original data is obtained through the formula *f*. *T* represents the characteristic sequence of the original sequence *X*, which is defined as $T=\{t_{1},t_{2},\ldots ,t_{k}\}$, where $t_{i}\in R^{l}$. The output of the encoder is used as the input of the decoder. The decoder reconstructs the original data according to the characteristic sequence *T*. The reconstructed data $Y=\{y_{1},y_{2},\ldots ,y_{k}\}$, where $y_{i}\in R^{d}$. The purpose of decoding is to verify whether the extracted features are valid. After the training of the AutoEncoder is completed, we only use the encoder to extract the characteristics of the original data to obtain more internal structure of the data. Figure 1 shows the basic structure of AutoEncoder. The encoding and decoding process follows the equations:

(1) $$t_{i}=f\left(w_{t}\cdot x_{i}+b_{t}\right),$$

(2) $$y_{i}=g\left(w_{y}\cdot t_{i}+b_{y}\right),$$

where $f\left(\cdot \right)$ and $g\left(\cdot \right)$ are the sigmoid functions, and $w_{t}$, $w_{y}$ and $b_{t}$, $b_{y}$ are weights and biases, respectively.

We train the AutoEncoder by minimizing reconstruction error

(3) $$L\left(X,Y\right)=\frac{1}{2}\sum_{i=1}^{n}{∥x_{i}-y_{i}∥}^{2}.$$

When the difference between the reconstructed data *Y* and the original data *X* is small enough, in other words, the output *T* of the coding process is valid, *T* is seen as the characteristics extracted from the original data.

In the urban traffic network, there is an interaction between the traffic flow at the current location and the traffic flow on surrounding roads. Therefore, we should consider not only the historical traffic flow data of the current location, but also the changes of upstream and downstream traffic flow. In this model, we use AutoEncoder to extract characteristics of upstream and downstream traffic flow data. Then, the extracted features are put into the prediction network to improve the accuracy of traffic flow prediction at the current location. Therefore, the input of AutoEncoder is the upstream and downstream traffic flow $X_{u}=\left\{x_{u1},x_{u2},\ldots ,x_{um}\right\}$ and $X_{d}=\left\{x_{d1},x_{d2},\ldots ,x_{dm}\right\}$, where $x_{ui},x_{di}\in R^{d}$. The characteristic sequence is defined as $Z_{t}=\left\{z_{1},z_{2},\ldots ,z_{m}\right\}$, where $z_{i}\in R^{l}$. In this paper, the processes of encoding and decoding follow the equations below:

(4) $$z_{i}=f\left(w_{z}\cdot \left(x_{ui}+x_{di}\right)+b_{z}\right),$$

(5) $$y_{i}=g\left(w_{y}\cdot z_{i}+b_{y}\right).$$

### 3.2. AE-LSTM Model

LSTM [43] has been widely used in many fields and achieved great success, such as in music generation, image caption, speech recognition and machine translation. LSTM improves the hidden-layer cell on the basis of RNN. The improvement of cell can make up for the gradient disappearance problem of RNN. LSTM adds some memory units, including forget gate, input gate and output gate. The memory units can further control the data and decide which should be retained and which should be deleted.

Since the upstream and downstream traffic flow will affect the traffic flow at the current location, we need to take these factors into account when predicting the current traffic flow. If all upstream and downstream traffic flow data are put into the prediction model, the data dimension will increase and the calculation will be too complicated. To solve this problem, we use the AutoEncoder to extract the characteristics of the upstream and downstream vehicle flow data. In other words, the dimensionality of the traffic flow data is reduced. The acquired characteristics are taken as a part of the input data of the prediction network. In this way, not only the impact of upstream and downstream traffic flow is considered, but also the data dimension is not increased too much. The input data of LSTM consists of two parts: the characteristics of upstream and downstream traffic flow data $z_{t}$ and the historical traffic flow data of the current position $x_{t}$.

**Figure 2:**
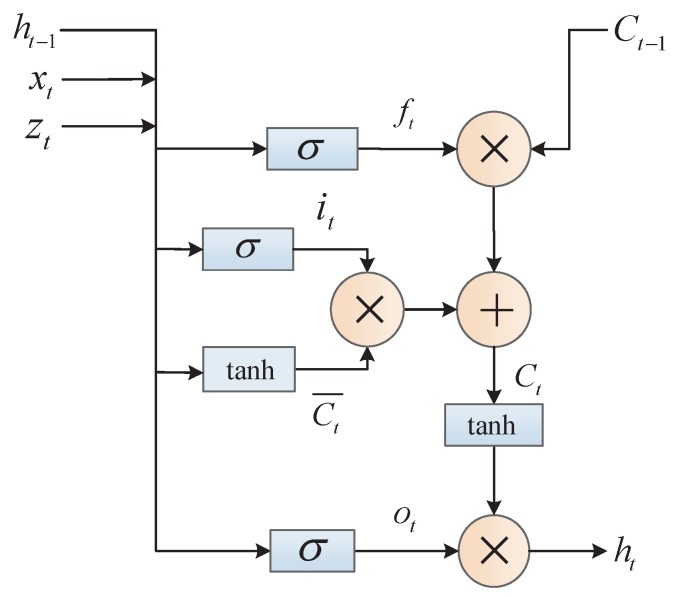
The structure of LSTM network. The input of the neuron is composed of $x_{t}$ and $z_{t}$, $x_{t}$ represents the traffic flow data of the current node, and $z_{t}$ represents the characteristic of the traffic flow data of the upstream and downstream.

The current position of the traffic flow data is expressed as $X=\{x_{1},x_{2},\ldots ,x_{m}\}$, where $x_{i}\in R^{d}$. The characteristics of upstream and downstream traffic flow data are represented by $Z_{t}$. The input information of the forgotten gate includes three parts: the current flow $x_{t}$, the upstream and downstream characteristic $z_{t}$, and the previous unit state $h_{t-1}$. The forgetting gate determines the information that should be discarded. The input information of the input gate is similar to that of the forgot gate. The input gate is used to select the information that should be input. $\bar{C_{t}}$ represents the input of the cell, which is added to the cell state. $C_{t}$ indicates that the status of the cell was updated. This step means removing part of the cell state at the previous time and adding part of the cell state at this time. The input information of the output gate is similar to that of the forgot gate. The output gate is used to select the information that should be output. Finally, the cell state is processed by tanh and multiplied by the output of output gate, among them $\sigma \left(x\right)=\frac{1}{1+e^{-x}}$ and $\tanh\left(x\right)=\frac{e^{x}-e^{-x}}{e^{x}+e^{-x}}$. The detailed structure of the LSTM is shown in Figure 2. The relevant formulas of AE-LSTM model are shown below:

(6) $$f_{t}=\sigma \left(w_{f1}\cdot x_{t}+w_{f2}\cdot z_{t}+w_{f3}\cdot h_{t-1}+b_{f}\right),$$

(7) $$i_{t}=\sigma \left(w_{i1}\cdot x_{t}+w_{i2}\cdot z_{t}+w_{i3}\cdot h_{t-1}+b_{i}\right),$$

(8) $$\bar{C_{t}}=\tanh\left(w_{c1}\cdot x_{t}+w_{c2}\cdot z_{t}+w_{c3}\cdot h_{t-1}+b_{c}\right),$$

(9) $$C_{t}=f_{t}\cdot C_{t-1}+i_{t}\cdot \bar{C_{t}},$$

(10) $$o_{t}=\sigma \left(w_{o1}\cdot x_{t}+w_{o2}\cdot z_{t}+w_{o3}\cdot h_{t-1}+b_{o}\right).$$

(11) $$h_{t}=o_{t}\cdot \tanh\left(C_{t}\right)$$

To consider the influence of upstream and downstream traffic flow but not increase the amount of calculation, we propose AE-LSTM prediction model, which combines the AutoEncoder and LSTM. Firstly, we use the AutoEncoder to extract the characteristics of original upstream and downstream traffic flow data. Then, we use LSTM model to predict the traffic flow. The specific prediction steps are shown below:

- The encoder of the AutoEncoder is used as the feature extractor to obtain the characteristics of upstream and downstream traffic flow data. The extracted features are put into the prediction network. Considering the influence of upstream and downstream on the traffic flow at the current location, the accuracy of traffic flow prediction can be improved.
- The characteristics of upstream and downstream traffic flow and the traffic flow data of the current position are combined as the input of LSTM. LSTM model predicts the traffic flow data at the next moment.

**Figure 3:**
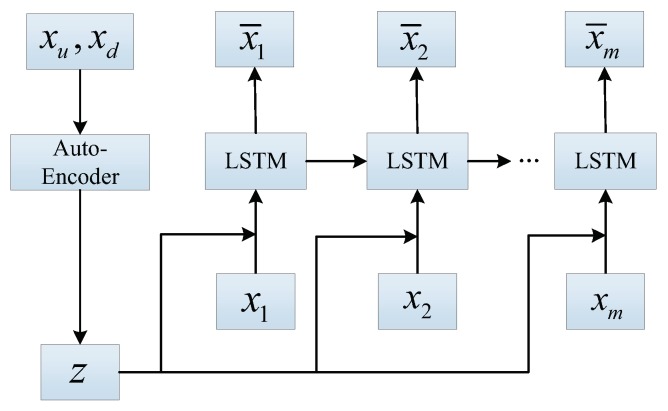
The structure of AE-LSTM for traffic flow prediction. AutoEncoder model is used to extract features and LSTM model is used to predict the traffic flow.

The AutoEncoder is a kind of unsupervised learning, which can be used as feature extractor of data. We determine the initial value of the weight matrix before the training. The weight matrix plays a very important role in the network. we hope to retain the characteristics of the original data after training the weight matrix. If the extracted feature can reconstruct the original data well, it indicates that the features of the original data can be effectively retained through the weight matrix. After the training of the AutoEncoder, we divide the AutoEncoder into two parts, which are the encoder and the decoder. The encoder, as a data feature extractor, is part of the AE-LSTM prediction model. The decoder is used to verify the validity of the extracted characteristics, and is discarded after the training. We attached a LSTM model after the encoder to form the AE-LSTM prediction model. The structure of the AE-LSTM model is shown in Figure 3.

In this paper, the Rectified Linear Unit (ReLU) function is selected as the activation function of the output layer in the whole network. The tanh function is selected as the activation function of other layers in the network. In this paper, the loss function is set to

(12) $$L\left(x,\bar{x}\right)=\frac{\sum_{n=1}^{N}{\left({\bar{x}}_{n}-x_{n}\right)}^{2}}{2N},$$

where $x_{n}$ represents the observed value, ${\bar{x}}_{n}$ represents the predicted value, and *N* represents the number of predicted values.

## 4. Model Implementation

The training of prediction network is one of the most important works, which is directly related to the final performance of prediction. We used the corresponding traffic flow data to train AutoEncoder and LSTM, respectively. Then, we fine-tuned the whole network and optimized the network parameters. The upstream and downstream traffic flow data $x_{u}$ and $x_{d}$ were put into AutoEncoder. We used back propagation to train parameters in AutoEncoder. The effective characteristics of traffic flow *Z* was obtained after encoding and decoding. In this way, the upstream and downstream traffic flow data could be considered in the prediction process, and the increasing of computational complexity could be avoided at the same time. The condition for the end of AutoEncoder training was that L(X,Y) was less than a threshold. L(X,Y) less than the threshold indicated that the decoder could reconstruct the original data through characteristic $Z_{t}$, and the error between the reconstructed traffic flow data and the original data was small enough. In other words, the extracted characteristics $Z_{t}$ was valid, and could reflect the internal structure of the original data. The characteristics $Z_{t}$ and the original data of current traffic flow $X_{t}$ were put into the network for training LSTM model. We still used the back propagation method to train the LSTM network. The AutoEncoder combined with LSTM to form AE-LSTM, and the whole network was fine-tuned. Algorithm 1 shows the pseudocode of AE-LSTM network training. The input data $X_{u}$, $X_{d}$ and $X_{t}$ represent upstream traffic flow data, downstream traffic flow data and current position traffic flow data, respectively.

Next, to further discuss the implementation of the model, we briefly describe the data processing, which is expressed as a matrix. The upstream and downstream traffic flow datasets are represented as

(13) $$X_{u}={\left[\begin{array}{cccc} X_{u1} & X_{u2} & \cdots & X_{um} \end{array}\right]}^{T}$$

and

(14) $$X_{d}={\left[\begin{array}{cccc} X_{d1} & X_{d2} & \cdots & X_{dm} \end{array}\right]}^{T},$$

where $X_{ui},X_{di}\in R^{d}$. After extracting the characteristics by AutoEncoder, we obtain the characteristics set of upstream and downstream vehicle flow

(15) $$Z_{t}={\left[\begin{array}{cccc} Z_{1} & Z_{2} & \cdots & Z_{m} \end{array}\right]}^{T},$$

where $Z_{i}\in R^{l},Z_{i}=\left\{z_{i1},z_{i2},\ldots ,z_{il}\right\}$. The historical prediction data $X_{t}$ are represented as

(16) $$X_{t}={\left[\begin{array}{cccc} X_{1} & X_{2} & \cdots & X_{m} \end{array}\right]}^{T},$$

where $X_{i}\in R^{d},X_{i}=\left\{x_{i1},x_{i2},\ldots ,x_{id}\right\}$. $X_{t}$ and $Z_{t}$ are combined to obtain a entire dataset as

(17) $$S_{t}={\left[\begin{array}{cccc} S_{1} & S_{2} & \cdots & S_{m} \end{array}\right]}^{T},$$

where $S_{i}\in R^{d+l},S_{i}=\left\{x_{i1},x_{i2},\ldots ,x_{id},z_{i1},z_{i2},\ldots ,z_{il}\right\}$. To accelerate the training and prediction process, we use the normalized method to process the original data. Before training the network, the original data are normalized. After obtaining the predicted results, the normal traffic flow predicted value can be obtained by reversing the normalization process.

**Algorithm 1** AE-LSTM prediction algorithm.**Input:** the training set $X_{u},X_{d},X_{t}$.**Output:** prediction result $\bar{X}$.
1:Use $X_{u}$ and $X_{d}$ to build the AutoEncoder training set $X_{AE}$.2:Initialize the weight matrices of AutoEncoder randomly.3:Put $X_{AE}$ into AutoEncoder.4:
**if** L(X,Y)<d **then**
5:  Calculate the error L(X,Y) by Equation (3).6:  Use the back propagation training the AutoEncoder.7:
**else**
8:  End the training.9:
**end if**
10:Generate the characteristics of upstream and downstream vehicle flow $Z_{t}$.11:**for**t=0 to epoch **do**12:  Put $Z_{t}$ and $X_{t}$ into the LSTM, and use Equations (6)–(11) for forward propagation.13:  Generate ${\bar{x}}_{t+1}=g\left(w_{z}\cdot h_{t}+b_{z}\right)$.14:  Calculate error.15:  Use the back propagation to update parameters.16:  Use forward propagation to update network status $h_{t}$, through Equations (6)–(11).17:
**end for**
18:Add LSTM after the encoder of AutoEncoder to form AE-LSTM.19:Fine-tuning the whole network, training initialization parameters.20:Input test data in AE-LSTM to generate the predicted value $\bar{X}$.21:Return $\bar{X}$.


## 5. Experimental and Analysis

### 5.1. Data Collection from Caltrans Performance Measurement System

**Figure 4:**
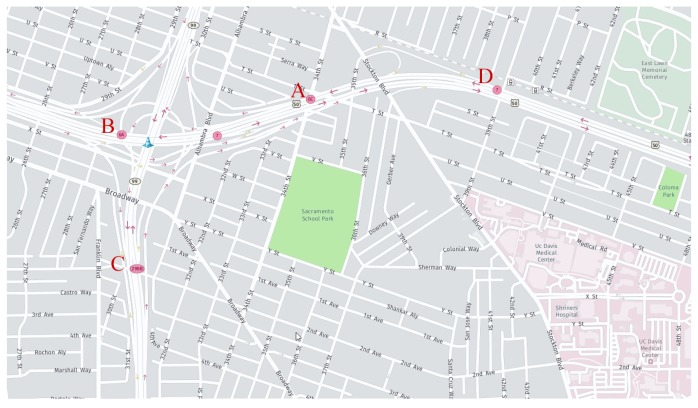
The data are collected around Sacramento County. The data collection stations are set up on the mainline of the four lanes.

In this experiment, traffic flow data were obtained from Caltrans Performance Measurement System (PeMS) [44]. PeMS is widely used by many researchers in experiments. To facilitate the comparison with other prediction algorithm, the dataset from PeMS was selected as the experimental data. PeMS is a professional traffic data acquisition system, in which 15,000 detectors have been deployed in California. The acquisition details of dataset are summarized in Table 1. The detectors record relevant traffic data every 30 s and store them in the database. PeMS aggregates the data to 5 min. The traffic flow is the number of vehicles passing through a detector over a period of time. For traffic flow, the 5-min data sample can be obtained by adding up the 30-s traffic data samples. Of course, the original data contain some missing or invalid data for various practical reasons. These invalid data have been reasonably estimated using estimation methods. To verify the performance of the AE-LSTM traffic flow prediction model, we applied it to the actual datasets. As shown in Figure 4, we obtained the statistical data recorded at several stations near Sacramento County. In Figure 4, the red dots represent the positions of data detection, which we marked as A, B, C, D, etc., and the red arrows represent the direction of traffic flow. In this study, the experiment was validated at multiple locations in Sacramento County,. Figure 4 only shows the actual location of a predicted location.

### 5.2. Experimental Setup

In this method, the AutoEncoder model extract the characteristics of the upstream and downstream traffic flow data, and the LSTM model predicts the traffic flow data. For the structure of the AE-LSTM network, we need to determine the number of input layers, the number of hidden layers, and the number of neurons in hidden layer. The input layer was set to 288×3. The number of hidden layers in the AutoEncoder model was 3, and the hidden neurons in each layer were 128, 128 and 32, respectively. The learning rate was set to 0.001. The number of hidden layers in LSTM model was 2, the number of neurons in each hidden layer was 128, the learning rate was set as 0.005, and the training number of epoch was 10. We obtained the traffic data from April to September 2018. The datasets was divided into two subsets, namely training set and test set. We used the first 176 days data of the six months in training process, which obtained the model. For LSTM model, the previous output was part of the input of the current iteration. Then, we used the generated model to predict the next seven days of data, and compared it to the last seven days of data during the six months. In addition, to obtain the impact of step ahead, we used the first five months data as training data, and predicted different steps ahead. Considering the time patterns of the traffic flow, we used the traffic flow data of the previous period to predict the data of the next moment. Considering the spatial pattern of traffic flow, we took the upstream and downstream traffic flow data as the input of the prediction model.

In this study, the prediction interval of traffic flow data was 5 min. To verify the performance of AE-LSTM prediction model, we chose the proposed algorithm [31], SVM model and CNN for comparison. All prediction models used the same datasets. In CNN model, we set input layer as 28×28, and convolutional layer as 20×20×20. Moreover, the pooling layer of CNN was set to 20×12×12, and learning rate was 0.1. We used ReLU as activation function in CNN. In SVM model, we used RBF as Kernel function. Moreover, after training, the regularization parameter was 7.5 and the Kernel function parameter was 0.54.

### 5.3. Model Evaluation

In the experiment, the performance of the traffic flow prediction models were measured by three indicators: Root Mean Square Error (RMSE), Mean Relative Error (MRE) and Mean Absolute Error (MAE). MRE is one of the most common indicators for comparing prediction accuracy. However, when the traffic flow value is large, the MRE will be small. Therefore, MAE and RMSE were used to supplement the error analysis. Their expressions are as follows:

(18) $$RMSE\left(\bar{x},x\right)=\sqrt{\frac{1}{N}\cdot \sum_{n=1}^{N}{\left({\bar{x}}_{n}-x_{n}\right)}^{2}},$$

(19) $$MRE\left(\bar{x},x\right)=\frac{1}{N}\cdot \sum_{n=1}^{N}\frac{|{\bar{x}}_{n}-x_{n}|}{x_{n}},$$

(20) $$MAE\left(\bar{x},x\right)=\frac{1}{N}\cdot \sum_{n=1}^{N}\left|{\bar{x}}_{n}-x_{n}\right|,$$

where ${\bar{x}}_{n}$ represents the predicted value, $x_{n}$ represents the observed value, and N represents the number of data.

### 5.4. Experimental Results

As shown in Table 2, we compared the errors of four prediction algorithms: CNN, SVM, the prediction algorithm in [17] and the AE-LSTM prediction algorithm. We compared the predict performance at different steps ahead. In general, the error of AE-LSTM prediction algorithm was smaller than the other compared algorithms. When the step ahead was 4, the MRE was 0.065. When the prediction of small step ahead was carried out, the performance of AE-LSTM algorithm had obvious advantage. When the prediction step ahead increased, the accuracy of prediction decreased slightly. In addition, the smaller was the prediction step ahead, the higher was the prediction accuracy and the better was the prediction performance. The reason for this phenomenon is that the multi-step prediction needs to use the previous prediction results. Unless the prediction is completely accurate, there must be an error between the predicted value and the observed value. Therefore, the larger is the step ahead of the prediction, the more errors of the previous prediction will be accumulated, leading to greater errors in the end. This is a common problem in the process of timing prediction. Therefore, as the prediction step size increased, the prediction accuracy of both AE-LSTM and the algorithm proposed in [17] increased, but the error of AE-LSTM increased less. It can be seen in Table 2 that the MRE of the two prediction algorithms were not significantly different. The reason is that the traffic flow was large, thus the difference between the predicted value and the observed value was relatively small. The difference between the predicted value and the observed value could be seen more intuitively from RMSE and MAE. Compared with the prediction algorithm in [17], RMSE and MAE of AE-LSTM had significant advantages.

**Figure 5:**
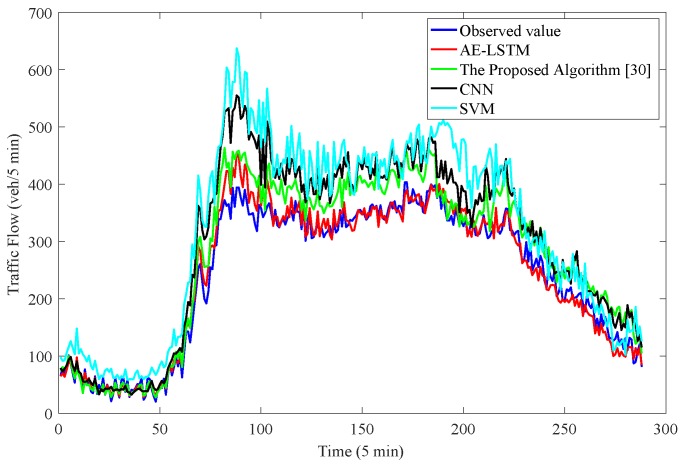
The blue line is the observation value on Sacramento County, the red line is the traffic flow prediction data obtained by AE-LSTM algorithm, and the green line represents the traffic flow prediction data obtained by the algorithm proposed in [17]. The black and light blue lines represent CNN and SVM, respectively.

As shown in Figure 5, we compared the observed value with the predicted results of four other prediction algorithms: CNN, SVM model, the algorithm proposed in [17] and the AE-LSTM prediction algorithm. The horizontal axis represents time, and every interval is 5 min. We predicted the traffic for 24 h, thus there are 288 data points. The vertical axis represents the number of traffic flows. In fact, the daily traffic pattern is similar. As shown in Figure 5, AE-LSTM had better prediction performance most of the time and matched well with the observed values. Compared with other prediction methods, AE-LSTM considered the influence of upstream and downstream traffic flow in the prediction process, it had better prediction performance at both night with less traffic flow and day with more traffic flow. The predictive performance of the algorithm proposed in [17] was better than that of CNN and SVM. Neither CNN nor SVM could well match observed values, but the overall prediction performance of SVM was worse.

**Figure 6:**
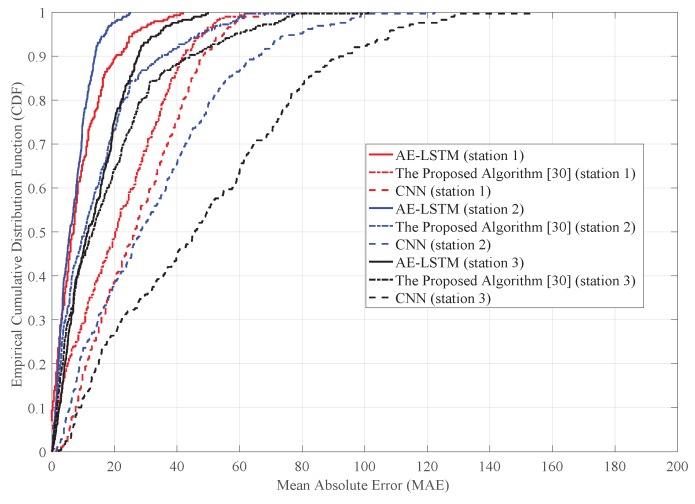
The cumulative distribution of MAE at different stations.

**Figure 7:**
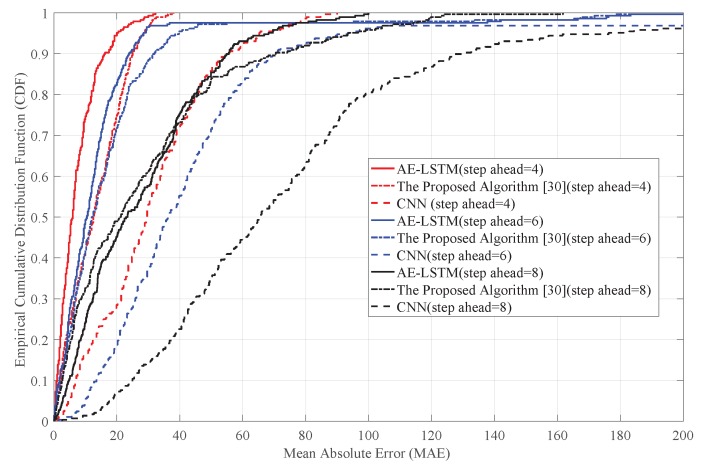
The cumulative distribution of MAE with different step-ahead.

Figure 6 and Figure 7 show the MAR cumulative distribution functions for each prediction method under different conditions. In other words, it presents the probability statistics of MAR for each prediction method. Figure 6 shows the MAE cumulative distribution of traffic flow prediction. The solid line represents the prediction error of AE-LSTM algorithm, the dotted line represents the prediction error of the algorithm proposed in [17], and the shorter dotted line represents the prediction error of CNN. For the AE-LSTM prediction model, the probability of MAE less than 25 was 90% at three different stations. The errors of AE-LSTM prediction model on different datasets were basically the same, which indicates that the prediction model has good robustness. For the algorithm proposed in [17] and CNN, the cumulative distribution of MAE at different stations varied greatly, which indicates that the performance of these methods are not stable for different datasets. For the algorithm proposed in [17], the probability of MAE less than 25 was 60%. For CNN, the probability of MAE being less than 25 was 30% at Stations 1 and 2, and 30% at Station 3. Overall, the prediction accuracy of CNN was not as high as that of the other two methods at the three stations. The prediction accuracy of AE-LSTM was higher than that of the algorithm proposed in [17] and CNN. The prediction error of the AE-LSTM and the prediction error of the algorithm proposed in [17] were different at Stations 1 and 2. At Station 3, AE-LSTM and the algorithm proposed in [17] had an almost equal probability of MAE less than 10. However, the probability of MAE less than 30 for the AE-LSTM prediction model was 95%, and the probability of MAE less than 25 for the algorithm proposed in [17] was less than 80%. Therefore, the prediction accuracy of AE-LSTM was higher than that of the algorithm proposed in [17].

Figure 7 shows the cumulative distribution of MAE at different step ahead. We predicted the traffic flow at Steps 4, 6, and 8, respectively. As shown in Figure 7, the error distribution trend of the two prediction algorithms was similar for different prediction steps ahead. As the step ahead increased, the errors of the three prediction methods increased. The reason for this phenomenon is that the circular neural network needs to use the data generated in the previous cycle in the prediction process. The larger is the step ahead, the lower is the accuracy of the data in the previous moment. As a result, the errors of the previous prediction are accumulated and MAE becomes bigger. When the step ahead is small, the prediction accuracy is high. That is because the data used in the prediction are mostly observed values. In addition, the performance of AE-LSTM model was obviously better when the prediction step ahead was 4 and 6. However, when the prediction step ahead was 8, the prediction accuracy of the three methods was not high because the prediction step ahead was too big.

**Figure 8:**
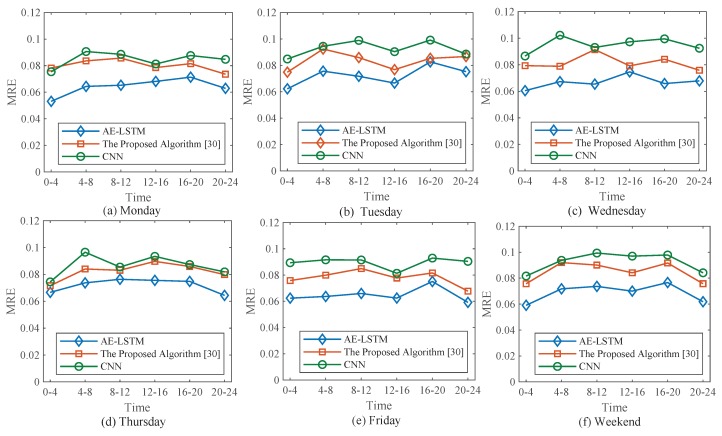
MRE statistics of the three prediction algorithms. We calculated the MAE of traffic flow prediction for six days.

To comprehensively evaluate the performance of the prediction algorithm in practical applications, we analyzed forecast data for six consecutive days. Figure 8 shows the prediction error for six days. To analyze the performance of the prediction algorithm, the MRE was calculated every 4 h. We choose two prediction methods for comparison. Among them, the CNN prediction model directly uses the historical data of traffic flow for prediction, excluding the upstream and downstream traffic flow data. As shown in Figure 8, the trend of daily prediction error was similar: MRE was smaller during 0–4 and 20–24, while MRE was larger during the other times. Among them, The MRE of AE-LSTM algorithm fluctuated around 0.065, the MRE of the algorithm proposed in [17] fluctuated around 0.08, and the MRE of CNN was higher and fluctuated around 0.09, and the maximum value even reached 0.1.

**Figure 9:**
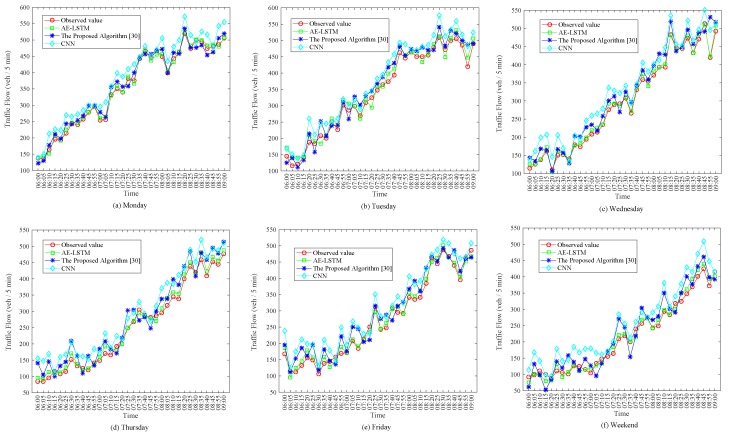
Observed and predicted values of traffic flow from 6:00 to 9:00.

Since the morning rush hour is from 6:00 to 9:00, the traffic flow may change greatly during this period, and traffic congestion and other problems often occur. To have more positive effects on traffic control, we analyzed the traffic flow data and forecast performance during this period. Figure 9 shows the observation values and forecast values of the traffic flow for six consecutive days between 6:00 and 9:00. As shown in Figure 9, the trend of daily traffic is similar. The traffic flow is 100 at 6:00, and the traffic flow fluctuation is 500 at 9:00. During this period, traffic flow fluctuates slightly, but the overall trend is increasing. We can see that the traffic flow on the weekend is less than that on the weekday. At 9:00 on the weekend, the traffic flow is about 400. In general, the predicted values of the AE-LSTM algorithm could better match the observed values and it had better prediction performance. AE-LSTM fully considers the influence of upstream and downstream traffic flow on the current position, thus the result is more stable and accurate.

## 6. Conclusions

In this paper, we propose an AE-LSTM prediction model to predict the traffic flow, which combines AutoEncoder and LSTM. The AE-LSTM prediction model not only considers the temporal characteristics but also uses the upstream and downstream data to capture the spatial characteristics of traffic flow. In addition, an AE-LSTM prediction algorithm is proposed. First, AutoEncoder and LSTM networks are trained, respectively, and then AE-LSTM is trained and fine-tuned. The algorithm is easy to implement and has good applicability. Finally, the performance of the AE-LSTM prediction model was verified by the real dataset from PeMS. Experimental results show that that AE-LSTM model had outstanding performance in traffic flow prediction. This study only considered time patterns and simple spatial patterns. In the future work, we will consider more complex spatial correlations and integrate them in neural networks.

**Data Availability:** In our experiment, we verified the performance of our prediction model on the dataset of PeMS [44].

## Figures and Tables

**Table 1:** The acquisition details of dataset.

Directional Distance	Controllers	Stations	Detectors	Traffic Census Stations	Features
41,236.0 mi	6943	18,350	45,170	16,527	Flow, occupancy and speed

**Table 2:** Performance comparison.

Algorithm	Step Ahead	Error Value		
		RMSE	MAE	MRE
AE-LSTM	4	26.32	16.15	0.065
	6	28.23	20.16	0.072
	8	76.87	43.15	0.131
The Proposed Algorithm [31]	4	35.45	25.26	0.088
	6	48.16	28.48	0.125
	8	99.52	52.86	0.161
CNN	4	46.22	34.59	0.011
	6	59.24	36.21	0.195
	8	105.16	59.86	0.198
SVM	4	49.54	39.02	0.023
	6	59.24	36.21	0.211
	8	107.36	62.86	0.209
